# Sexually transmitted infections among women randomised to depot medroxyprogesterone acetate, a copper intrauterine device or a levonorgestrel implant

**DOI:** 10.1136/sextrans-2020-054590

**Published:** 2020-11-18

**Authors:** Jennifer Deese, Neena Philip, Margaret Lind, Khatija Ahmed, Joanne Batting, Mags Beksinska, Vinodh A Edward, Cheryl E Louw, Maricianah Onono, Thesla Palanee-Phillips, Jennifer A Smit, Jared M Baeten, Deborah Donnell, Timothy D Mastro, Nelly R Mugo, Kavita Nanda, Helen Rees, Charles Morrison

**Affiliations:** 1 Global Public Health Impact Center, RTI International, Research Triangle Park, North Carolina, USA (formerly with FHI 360); 2 Mailman School of Public Health, Columbia University, New York, New York, USA; 3 Department of Global Health, International Clinical Research Center, University of Washington, Seattle, Washington, USA; 4 Setshaba Research Centre, Pretoria, South Africa; 5 Effective Care Research Unit, University of the Witwatersrand, East London, South Africa; 6 MatCH Research Unit, Department of Obstetrics and Gynaecology, Faculty of Health Sciences, University of the Witwatersrand, Durban, South Africa; 7 The Aurum Institute, Johannesburg, South Africa; 8 School of Pathology, Faculty of Health Sciences, University of the Witwatersrand, Johannesburg, South Africa; 9 Madibeng Centre for Research, Brits, South Africa; 10 Faculty of Health Sciences, University of Pretoria, Pretoria, South Africa; 11 Center for Microbiology Research, Kenya Medical Research Institute, Nairobi, Kenya; 12 Faculty of Health Sciences, Wits Reproductive Health and HIV Institute, Johannesburg, South Africa; 13 Global Health, Medicine, and Epidemiology, University of Washington, Seattle, Washington, USA; 14 Vaccine and Infectious Disease Division, Fred Hutchinson Cancer Research Center, Seattle, Washington, USA; 15 FHI 360, Durham, North Carolina, USA; 16 Department of Global Health, University of Washington, Seattle, Washington, USA; 17 Center for Clinical Research, Kenya Medical Research Institute, Nairobi, Kenya; 18 Product Development and Introduction, FHI 360, Durham, North Carolina, USA; 19 Wits Reproductive Health & HIV Institute, University of the Witwatersrand, Johannesburg, South Africa; 20 Behavioral, Epidemiological and Clinical Sciences, FHI 360, Durham, North Carolina, USA

**Keywords:** contraception, clinical trials, chlamydia trachomatis, neisseria gonorrhoeae

## Abstract

**Objectives:**

Reproductive aged women are at risk of pregnancy and sexually transmitted infections (STI). Understanding drivers of STI acquisition, including any association with widely used contraceptives, could help us to reduce STI prevalence and comorbidities. We compared the risk of STI among women randomised to three contraceptive methods.

**Methods:**

We conducted a secondary analysis to assess the risk of chlamydia and gonorrhoea in a clinical trial evaluating HIV risk among 7829 women aged 16–35 randomised to intramuscular depot medroxyprogesterone acetate (DMPA-IM), a copper intrauterine device (IUD) or a levonorgestrel (LNG) implant. We estimated chlamydia and gonorrhoea prevalences by contraceptive group and prevalence ratios (PR) using log-binomial regression.

**Results:**

At baseline, chlamydia and gonorrhoea prevalences were 18% and 5%, respectively. Final visit chlamydia prevalence did not differ significantly between DMPA-IM and copper IUD groups or between copper IUD and LNG implant groups. The DMPA-IM group had significantly lower risk of chlamydia compared with the LNG implant group (PR 0.83, 95% CI 0.72 to 0.95). Final visit gonorrhoea prevalence differed significantly only between the DMPA-IM and the copper IUD groups (PR 0.67, 95% CI 0.52 to 0.87).

**Conclusions:**

The findings suggest that chlamydia and gonorrhoea risk may vary with contraceptive method use. Further investigation is warranted to better understand the mechanisms of chlamydia and gonorrhoea susceptibility in the context of contraceptive use.

## Background

Reproductive aged women are at risk of both pregnancy and sexually transmitted infections (STI). The modern contraceptive prevalence among married and unmarried women in South Africa is 54% and 64%, respectively, with injectable progestins being most widely used.^1^ Moreover, current global efforts aim towards all women having access to a range of reliable contraceptives options.^2^ The prevalences of chlamydia and gonorrhoea are high among women in Africa, particularly among younger women. A recent meta-analysis of over 37 000 women estimated prevalences for chlamydia and gonorrhoea by region and population type (South Africa clinic/community-based, Eastern Africa higher-risk and Southern/Eastern Africa clinic community-based). High chlamydia and gonorrhoea prevalences were found among 15–24 year-old South African women and high risk populations in East Africa.^3^ Both chlamydia and gonorrhoea are associated with numerous comorbidities including pelvic inflammatory disease (PID), ectopic pregnancy, infertility, increased risk of HIV and other STIs, as well as significant social harm.^4^

While STIs are a significant global health burden, data on STI prevalence by gender and drivers of infection are limited, hindering an effective public health response.^5^ Moreover, data on the association between contraceptive use and risk of non-HIV STIs are limited. The WHO recently reported stagnation in efforts to decrease global STI incidence.^5^ Understanding drivers of STI acquisition, including any possible associations with widely used contraceptive methods, is necessary to effectively target public health responses that reduce STI incidence and associated comorbidities.

The ECHO Trial (ClinicalTrials.gov Identifier: NCT02550067) was a multicentre, open-label randomised trial of 7829 HIV-seronegative women seeking effective contraception in Eswatini, Kenya, South Africa and Zambia; detailed trial methods and results have been published.^6 7^ We conducted a secondary analysis of ECHO trial data to evaluate absolute and relative chlamydia and gonorrhoea final visit prevalences among women randomised to intramuscular depot medroxyprogesterone acetate (DMPA-IM), a copper intrauterine device (IUD) and a levonorgestrel (LNG) implant.

## Methods

### Study design, participants and ethics

Women were enrolled in the ECHO trial from December 2015 through September 2017. Institutional review boards at each site approved the study protocol and women provided written informed consent before any study procedures. In brief, women who were not pregnant, HIV-seronegative, aged 16–35 years, seeking effective contraception, without medical contraindications, willing to use the assigned method for 18 months, reported not using injectable, intrauterine or implantable contraception for the previous 6 months and reported being sexually active, were enrolled. At every visit, participants received HIV risk reduction counselling, HIV testing and STI management, condoms and, as it became a part of national standard of care, HIV pre-exposure prophylaxis. Counselling messages related to HIV risk were implemented consistently across the three groups throughout the trial.^6^

The trial was implemented in accordance with the Declaration of Helsinki and Good Clinical Practice. Informed consent was obtained from participants or their parents/guardians and human experimentation guidelines of the United States Department of Health and Human Services and those of the authors' institution(s) were followed.

### Contraceptive exposure

At enrolment, women were randomly assigned (1:1:1) to DMPA-IM, copper IUD or LNG implant.^6^ Participants received an injection of 150 mg/mL DMPA-IM (Depo Provera; Pfizer, Puurs, Belgium) at enrolment and every 3 months until the final visit at 18 months after enrolment, a copper IUD (Optima TCu380A; Injeflex, Sao Paolo, Brazil) or a LNG implant (Jadelle; Bayer, Turku, Finland) at enrolment. Women returned for follow-up visits at 1 month after enrolment to address initial contraceptive side-effects and every 3 months thereafter, for up to 18 months with later enrolling participants contributing 12 to 18 months of follow-up. Visits included HIV serological testing, contraceptive counselling, syndromic STI management and safety monitoring.

### STI outcomes

The primary outcomes of this secondary analysis were prevalent chlamydia and gonorrhoea infection at the final visit. Syndromic STI management was provided at screening and all follow-up visits. Nucleic acid amplification testing (NAAT) for *Chlamydia trachomatis* and *Neisseria gonorrhoeae* was conducted at screening and final visits, at the visit of HIV detection for participants who became HIV infected and at clinical discretion. Any untreated participants with positive NAAT results were contacted to return to the study clinic for treatment.

### Covariates

At baseline (inclusive of screening and enrolment visits), we collected demographic, sexual and reproductive risk behaviour and reproductive and contraceptive history data. Baseline risk factors evaluated as covariates included age, whether the participant earned her own income, chlamydia and gonorrhoea status, herpes simplex virus type 2 (HSV-2) sero-status and suspected PID. Final visit factors evaluated as covariates included number of sex partners in the past 3 months, number of new sex partners in the past 3 months, HIV serostatus, HSV-2 serostatus, condom use in the past 3 months, sex exchanged for money/gifts, sex during vaginal bleeding, follow-up time and number of pelvic examinations during follow-up. Age and HSV-2 serostatus were evaluated for effect measure modification.

### Statistical analysis

We conducted analyses using R V.3.5.3 (Vienna, Austria), and log-binomial regression to estimate chlamydia and gonorrhoea prevalences within each contraceptive group and pairwise prevalence ratios (PR) between each arm in as-randomised and consistent use analyses.

In the as-randomised analysis, we analysed participants by the contraceptive method assigned at randomisation independent of method adherence. We estimated crude point prevalences by arm and study site and pairwise adjusted PRs.

In the consistent use analysis, we only included women who initiated use of their randomised contraceptive method and maintained randomised method adherence throughout follow-up. We estimated crude point prevalences by arm and pairwise adjusted PRs, with evaluation of age and HSV-2 status first as potential effect measure modifiers, and all covariates above as potential confounders. Study site and age were retained in the final model; other covariates were retained if their inclusion in the base model led to a 10% change in the effect estimate through backwards selection.

### Supplementary analyses

Additional supporting analyses to assess postrandomisation potential sources of bias were conducted to inform interpretation of results. These include evaluation of recent sexual behaviour at enrolment, month 9 and the final visit; cohort participation (ie, follow-up time, early discontinuation and timing of randomised method discontinuation) and health outcomes (ie, final visit HIV and HSV-2 status) and frequency and results of pelvic examinations by STI status, site and visit month by randomised arm.

## Results

**Figure 1:**
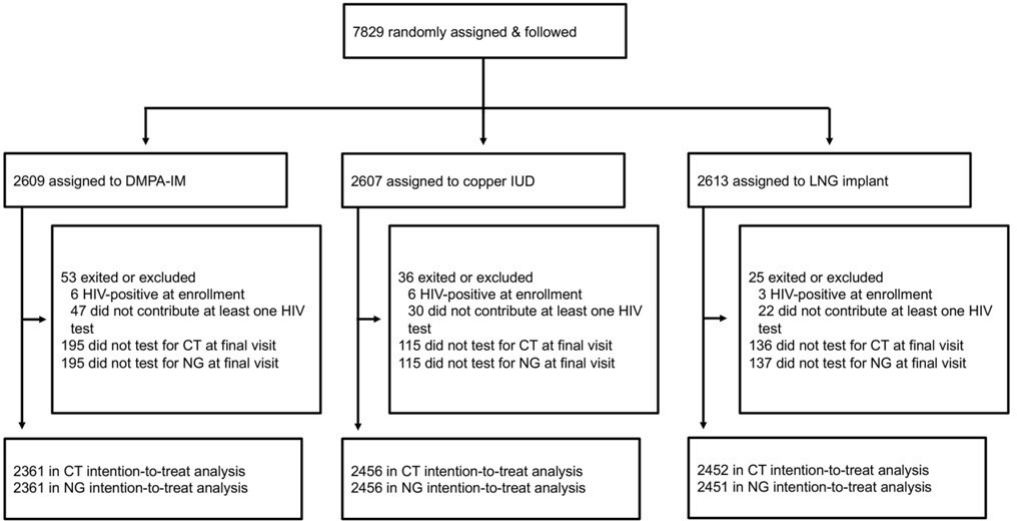
Study profile. DMPA-IM, depot medroxy progesterone acetate; IUD, intrauterine device; LNG, levonorgestrel.

A total of 7829 women were randomly assigned as follows: 2609 to the DMPA-IM group, 2607 to the copper IUD group and 2613 to the LNG implant group (figure 1). Participants were excluded if they were HIV positive at enrolment, did not have at least one HIV test or did not have chlamydia and gonorrhoea test results at the final visit. Overall, 90%, 94% and 93% from the DMPA-IM, copper IUD and LNG implant groups, respectively, were included in analyses.

### Participant characteristics

Baseline characteristics were similar across groups (table 1). Nearly two-third of enrolled women (63%) were aged 24 and younger and 5768 (74%) of the study population resided in South Africa.

**Table 1:** Participant baseline and final visit characteristics

	Total (n=7829)	DMPA-IM (n=2609)	Copper IUD (n=2607)	LNG implant (n=2613)
Baseline	n (%)	n (%)	n (%)	n (%)
Age				
16–24 years old	4967 (63)	1673 (64)	1627 (62)	1667 (64)
25–35 years old	2862 (37)	936 (36)	980 (38)	946 (36)
Highest education level				
No schooling	49 (<1)	16 (<1)	12 (1)	21 (1)
Primary school (not complete)	400 (5)	118 (5)	139 (5)	143 (6)
Primary school (complete)	323 (4)	98 (4)	108 (4)	117 (5)
Secondary school (not complete)	2866 (37)	975 (37)	956 (37)	935 (36)
Secondary school (complete)	2949 (38)	992 (38)	974 (37)	983 (38)
Attended postsecondary school	1242 (16)	410 (16)	418 (16)	414 (16)
Earns own income	1697 (22)	565 (22)	565 (22)	567 (22)
Study area				
Eswatini	502 (6)	167 (6)	167 (6)	168 (6)
Kenya	901 (12)	299 (12)	301 (12)	301 (12)
South Africa (Province)				
Eastern Cape	614 (8)	205 (8)	205 (8)	204 (8)
Gauteng	2062 (26)	686 (26)	687 (26)	689 (26)
KwaZulu-Natal	2125 (27)	711 (27)	707 (27)	707 (27)
North West	407 (5)	136 (5)	134 (5)	137 (5)
Western Cape	560 (7)	187 (7)	186 (7)	187 (7)
Zambia	658 (8)	218 (8)	220 (8)	220 (8)
Sexual and reproductive history				
Nulligravid	1462 (19)	509 (20)	486 (19)	467 (18)
No prior contraceptive use	586 (8)	206 (8)	184 (7)	196 (8)
Baseline STI prevalence				
Chlamydia trachomatis*	1420 (18)	454 (17)	486 (19)	480 (18)
Neisseria gonorrhoeae*	368 (5)	117 (5)	124 (5)	127 (5)
HSV-2†	3789 (48)	1271 (49)	1282 (49)	1236 (47)
**Final visit**	**n (%**)	**n (%**)	**n (%**)	**n (%**)
Mean number months follow-up (SD)	16 (5.0)	15 (5.6)	16 (4.7)	16 (4.7)
Randomised method non-adherence‡	1468 (19)	675 (26)	477 (18)	316 (12)
Method discontinuation (N)§	1364	674	384	306
Timing of first method discontinuation§				
Enrolment—6 months	446 (5.7)	164 (6.3)	176 (6.8)	106 (4.1)
7–12 months	644 (8.2)	383 (14.7)	130 (5.0)	131 (5.0)
13–18 months¶	274 (3.5)	127 (4.9)	78 (3.0)	69 (2.6)

*Twelve participants did not have available baseline *C. trachomatis* or *N. gonorrhoeae* results.

†An HSV-2 enzyme immunoassay index value of less than 0.90 was classified as negative, 0.90–3.50 as indeterminate and more than 3.50 as positive.

‡Includes women who did not receive randomised method at baseline visit or who discontinued randomised method during follow-up. Method discontinuation was defined as either use of a secondary hormonal method or, in the DMPA-IM group, more than 119 days between injections; in the Cu-IUD group, a spontaneous, complete expulsion without reinsertion within 28 days or IUD removal without reinsertion on the same calendar day or in the LNG implant group, implant removal without reinsertion on the same calendar day.

§A subset of randomised method non-adherence, as defined above.

¶Timing of method discontinuation could have occurred during the 18th month of follow-up but prior to study discontinuation at the scheduled 18th month visit.

DMPA-IM, intramuscular depot medroxyprogesterone acetate; HSV-2, herpes simplex virus type 2; IUD, intrauterine device; LNG, levonorgestrel.

The duration of participation averaged 16 months with no differences between randomised groups (table 1). A total of 1468 (19%) women either did not receive their randomised method or discontinued use during follow-up. Overall method continuation rates were high with minimal differences between randomised groups when measured by person-years.^6^ The proportion, however, of method non-adherence as defined in this analysis (ie, did not receive randomised method at baseline or discontinued randomised method at any point during follow-up), was greater in the DMPA-IM group (26%), followed by the copper IUD (18%) and LNG implant (12%) groups. Timing of discontinuation also differed across methods. During the first 6 months, method discontinuation was highest in the copper IUD group (7%) followed closely by DMPA-IM (6%) and LNG implant (4%) groups. Between 7 and 12 months of follow-up, it was highest in DMPA-IM group (15%), with equivalent proportions in the LNG implant (5%) and copper IUD (5%) groups.

### Point prevalences of chlamydia and gonorrhoea at baseline and final visits

**Figure 2:**
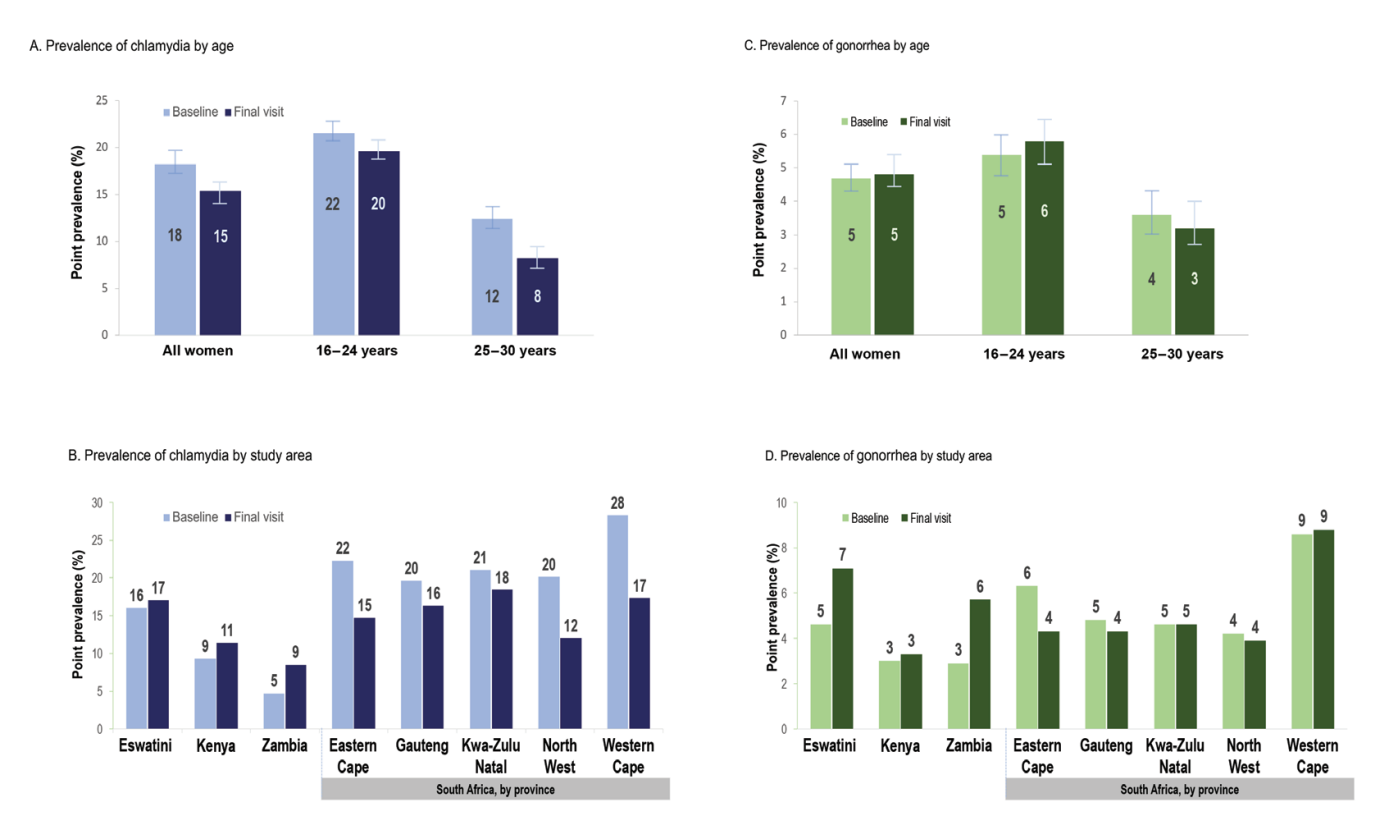
Point prevalence (per 100 persons) of chlamydia and gonorrhoea at baseline and final visit by age category and study site region. Y-axis scale differs for chlamydia and gonorrhoea figures.

In total, 18% of women had chlamydia at baseline (figure 2A) and 15% at the final visit. Among women 24 years and younger, 22% and 20% had chlamydia at baseline and final visits, respectively. Women aged 25–35 at baseline were less likely to have chlamydia at both baseline (12%) and final visits (8%) compared with younger women. Baseline chlamydia prevalence ranged from 5% in Zambia to 28% in the Western Cape, South Africa (figure 2B).

Among all women, 5% had gonorrhoea at baseline and the final visit (figure 2C). Women aged 24 and younger were more likely to have gonorrhoea compared with women aged 25 and older at both baseline (5% vs 4%, respectively) and the final visit (6% vs 3%, respectively). Baseline gonorrhoea prevalence ranged from 3% in Zambia and Kenya to 9% in the Western Cape, South Africa (figure 2D). Similar prevalences were observed at the final visit.

### Point prevalences of chlamydia and gonorrhoea at final visit by randomised contraceptive method

Fourteen per cent of women randomised to DMPA-IM, 15% to copper IUD and 17% to LNG implant had chlamydia at the final visit (table 2).

**Table 2:** *Chlamydia* trachomatis and *Neisseria gonorrhoeae* prevalence at final visit

	DMPA-IM group		Copper IUD group		LNG implant group		DMPA-IM vs copper IUD		DMPA-IM vs LNG implant		Copper IUD vs LNG implant	
	Events/ women	PP (95% CI)	Events/ women	PP (95% CI)	Events/ women	PP (95% CI)	PR (95% CI)	P value	PR (95% CI)	P value	PR (95% CI)	P value
*Chlamydia trachomatis*												
As randomised†	327/2361	13.85 (12.52 to 15.32)	378/2456	15.39 (14.03 to 16.89)	412/2452	16.80 (15.39 to 18.35)	0.9 (0.79 to 1.04)	0.144	0.83 (0.72 to 0.95)	0.005	0.92 (0.81 to 1.04)	0.178
Continuous use‡	221/1686	13.11 (11.59 to 14.82)	309/1995	15.49 (13.98 to 17.16)	377/2139	17.63 (16.08 to 19.32)	0.86 (0.74 to 1.01)	0.062	0.77 (0.66 to 0.89)	0.001	0.89 (0.78 to 1.02)	0.093
*Neisseria gonorrhoeae*												
As randomised†	91/2361	3.85 (3.15 to 4.71)	141/2456	5.74 (4.89 to 6.74)	120/2451	4.90 (4.11 to 5.83)	0.67 (0.52 to 0.87)	0.002	0.79 (0.61 to 1.03)	0.085	1.18 (0.93 to 1.49)	0.175
Continuous use‡	64/1686	3.80 (2.99 to 4.83)	125/ 1995	6.27 (5.29 to 7.42)	110/2138	5.14 (4.29 to 6.17)	0.67 (0.49 to 0.90)	0.007	0.75 (0.56 to 1.02)	0.064	1.13 (0.87 to 1.46)	0.318

*P value is for whether PR differs by subgroup. Subgroup-specific PRs are provided with 95% CIs.

†Adjusted for site only.

‡*C. trachomatis* analysis: adjusted for age group and baseline *C. trachomatis* status; *N. gonorrhoeae* analysis: adjusted for final visit HIV status and total number of pelvic examinations during the study. *C. trachomatis* analysis: adjusted for age group and baseline *C. trachomatis* status; *N. gonorrhoeae* analysis: adjusted for final visit HIV status and total number of pelvic exams during the study.

DMPA-IM, intramuscular depot medroxyprogesterone acetate; IUD, intrauterine device; LNG, levonorgestrel; PP, point prevalence; PR, prevalence ratio.;

The prevalence of chlamydia did not significantly differ between DMPA-IM and copper IUD groups (PR 0.90, 95% CI (0.79 to 1.04)) or between copper IUD and LNG implant groups (PR 0.92, 95% CI (0.81 to 1.04)). Women in the DMPA-IM group, however, had a significantly lower risk of chlamydia compared with the LNG implant group (PR: 0.83, 95% CI (0.72 to 0.95)). Findings from the consistent use analysis were similar, and neither age nor HSV-2 status modified the observed associations.

Four per cent of women randomised to DMPA-IM, 6% to copper IUD and 5% to LNG implant had gonorrhoea at the final visit (table 2). Gonorrhoea prevalence did not significantly differ between DMPA-IM and LNG implant groups (PR: 0.79, 95% CI (0.61 to 1.03)) or between copper IUD and LNG implant groups (PR: 1.18, 95% CI (0.93 to 1.49)). Women in the DMPA-IM group had a significantly lower risk of gonorrhoea compared with women in the copper IUD group (PR: 0.67, 95% CI (0.52 to 0.87)). Results from as randomised and continuous use analyses did not differ; and again, neither age nor HSV-2 status modified the observed associations.

### Clinical assessment by randomised contraceptive method

To assess the potential for outcome ascertainment bias, we evaluated the frequency of pelvic examinations and abdominal/pelvic pain and discharge by study arm. Women in the copper IUD group were generally more likely to receive a pelvic examination during follow-up as compared with women in the DMPA-IM and LNG implant groups (online supplemental appendix 1). Similarly, abdominal/pelvic pain on examination or abnormal discharge was observed most frequently in the copper IUD group. The number of pelvic examinations met the prespecified criteria for retention in the adjusted gonorrhoea model but not in the chlamydia model.

10.1136/sextrans-2020-054590.supp1 Supplementary data

### Frequency of syndromic symptoms and potential reinfection

**Figure 3:**
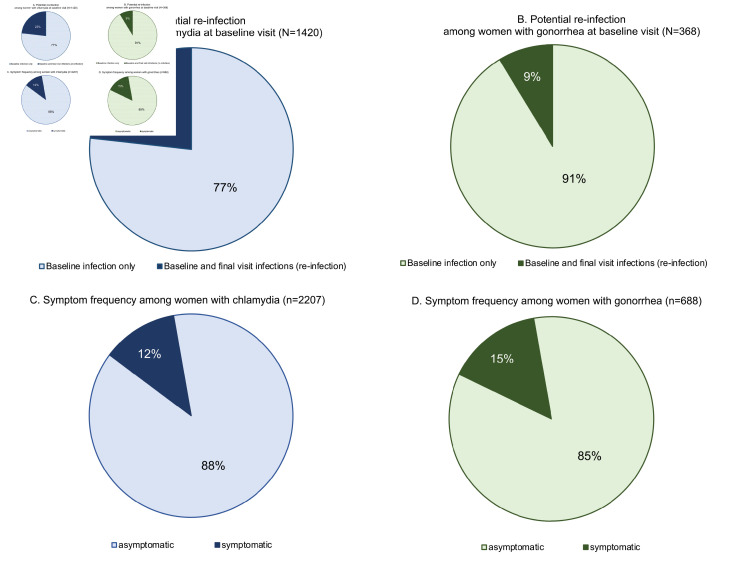
Potential reinfection and symptoms among women with chlamydia or gonorrhoea. Data are pooled across the screening and final visits in figures (C) and (D). Symptomatic is defined as presenting with abnormal vaginal discharge and/or abdominal/pelvic pain. Final visit infection is described as potential reinfection because test of cure was not conducted following baseline diagnosis and treatment.

Among women who had chlamydia at baseline, 23% were also positive at the final visit (online supplemental appendix 2, figure 3A). Nine per cent of gonorrhoea-positive women at baseline were also positive at the final visit (online supplemental appendix 2, figure 3B). Across both baseline and final visits, a minority of women with chlamydia or gonorrhoea presented with signs and/or symptoms. Among chlamydia-positive women, only 12% presented with either abnormal vaginal discharge and/or abdominal/pelvic pain at their test-positive visit (online supplemental appendix 2, figure 3C). Similarly, only 15% of gonorrhoea-positive women presented with abnormal vaginal discharge and/or abdominal/pelvic pain at their test-positive visit (online supplemental appendix 2, figure 3D).

## Discussion

We observed differences in final prevalences of chlamydia and gonorrhoea by contraceptive group in both as-randomised and consistent-use analyses. The DMPA-IM group had lower final visit chlamydia and gonorrhoea prevalences as compared with copper IUD and LNG implant groups, though only the DMPA-IM versus the copper IUD comparison of gonorrhoea and DMPA-IM versus LNG implant comparison of chlamydia reached statistical significance. These are novel findings that have not previously been reported to our knowledge and were determined in a randomised trial setting with high participant retention, robust biomarker testing and high randomised method adherence. Interestingly, the copper IUD group had higher gonorrhoea and lower chlamydia prevalence compared with the LNG implant group, though neither finding was statistically significant.

Two recent systematic reviews of the association between contraceptives and STIs found inconsistent and insufficient evidence on the association between the contraceptive methods under study in ECHO and chlamydia and gonorrhoea.^8 9^ Neither systematic review identified any randomised studies or any direct comparative evidence for DMPA-IM, copper IUD and LNG implant, thus enabling a unique scientific contribution from this secondary trial analysis. Nonetheless, these findings should be interpreted in light of biological plausibility, as well as the design strengths and limitations of this analysis.

The emerging science on the biological mechanisms underlying HIV susceptibility demonstrates the complex relationship between the infectious pathogen, the host innate and adaptive immune response and the interaction of both with the vaginal microbiome and other -omes. Data on these factors in relationship to chlamydia and gonorrhoea acquisition are much more limited but can be assumed to be equally complex. Vaginal microbiome composition, including microbial metabolic by-products, have been shown to significantly modify risk of HIV acquisition and to vary with exogenous hormone exposure, menstrual cycle phase, ethnicity and geography.^10–12^ These same biological principles likely apply to chlamydia and gonorrhoea susceptibility. While DMPA-IM has been associated with decreased bacterial vaginosis (BV), initiation of the copper IUD has been associated with increased BV prevalence, and BV is associated with chlamydia and gonorrhoea acquisition.^13 14^ Moreover, *Lactobacillus crispatus*, which is less abundant in BV, has been shown to inhibit HeLa cell infection by *Chlamydia trachomatis* and inhibits growth of *Neisseria gonorrhoeae* in animal models.^15 16^ In addition, microbial community state types that are deficient in *Lactobacillus crispatus* and/or dominated by dysbiotic species are associated with inflammation, which is a driver of both STI and HIV susceptibility. Thus, while the exact mechanisms of chlamydia and gonorrhoea infection in the presence of exogenous hormones and varying host microbiomes are unknown, it is biologically plausible that these complex factors may result in differential susceptibility to chlamydia and gonorrhoea among DMPA-IM, copper IUD and LNG implant users.

An alternative explanation for these findings may be postrandomisation differences in clinical care and/or sexual behaviour. Participants in the copper IUD arm were more likely to have pelvic examinations and more likely to have discharge compared with women in the DMPA-IM and LNG implant groups. While interim STI testing and/or treatment were not documented, women in the copper IUD arm may have been more likely to receive syndromic STI treatment during follow-up due to more examination and observed discharge. More frequent STI treatment in the copper IUD group would theoretically lower the final visit point prevalence relative to women in the DMPA-IM and LNG implant arms, suggesting that the observed lower risk of STI in the DMPA-IM arm is not due to differential examination, testing and treatment. Differential sexual risk behaviour may also have influenced the results. As reported previously, women in the DMPA-IM group less frequently reported condomless sex and multiple partners than women in the other groups, and both DMPA-IM and LNG implant users less frequently reported new partners and sex during menses than copper IUD users.^6^ Statistical control of self-reported sexual risk behaviour in the consistent-use analysis may have been inadequate if self-reported sexual behaviour was inaccurately or insufficiently reported.

A second alternative explanation may be differences in randomised method non-adherence, which was greater in the DMPA-IM group, compared with copper IUD and LNG implant groups. Yet, the consistency of findings in the as-randomised and continuous use analyses suggests that method non-adherence had minimal effect on study outcomes. Taken as a whole, these findings indicate that there may be real differences in chlamydia and gonorrhoea risk associated with use of DMPA-IM, the copper IUD and LNG implant. However, any true differential risk by method must be evaluated in light of the holistic benefits and risks of each method.

The high observed chlamydia and gonorrhoea prevalences, despite intensive counselling and condom provision, warrants attention, particularly among women ages 24 years and younger and among women in South Africa and Eswatini. While the ECHO study was conducted in settings of high HIV/STI incidence, enrolment criteria did not purposefully target women at highest risk of HIV/STI in the trial communities, suggesting that the observed prevalences may be broadly applicable to women seeking effective contraception in those settings. Improved approaches are needed to prevent STIs, including options for expedited partner treatment, to prevent reinfection.

As expected, few women testing positive for chlamydia or gonorrhoea presented with symptoms (12% and 15%, respectively), and a substantial proportion of women who were positive and treated at baseline were infected at the final visit despite syndromic management during the follow-up. Given that syndromic management is the standard of care within primary health facilities in most trial settings, these data suggest that a large proportion of infection among reproductive aged women is missed, exacerbating the burden of curable STIs and associated morbidities. Routine access to more reliable diagnostics, like NAAT and novel point-of-care diagnostic tests, will be key to managing asymptomatic STIs and reducing STI prevalence and related morbidities in these settings.^17^

This secondary analysis of the ECHO trial has strengths and limitations. Strengths include the randomised design with comparator groups of equal STI baseline risk. Participants had high adherence to their randomised contraceptive method.^6^ While all participants received standardised clinical care and counselling, the unblinded randomisation may have allowed postrandomisation differences in STI risk over time by method. It is possible that participants modified their risk-taking behaviour based on study counselling messages regarding the potential association between DMPA-IM and HIV.

In conclusion, our analyses suggest that DMPA-IM users may have lower risk of chlamydia and gonorrhoea compared with LNG implant and copper IUD users, respectively. Further investigation is warranted to better understand the mechanisms of chlamydia and gonorrhoea susceptibility in the context of contraceptive use. Moreover, the high chlamydia and gonorrhoea prevalences in this population, independent of contraceptive method, warrants urgent attention.

**Key messages**

- The prevalence of chlamydia and gonorrhoea varied by contraceptive method in this randomised trial.
- High chlamydia and gonorrhoea prevalences, despite intensive counselling and condom provision, warrants attention, particularly among young women in South Africa and Eswatini.
- Most chlamydia and gonorrhoea infections were asymptomatic; therefore, routine access to reliable diagnostics are needed to effectively manage and prevent STIs in African women.

## Data Availability

Data are available on reasonable request. As of the time of publication, data access applications are in process with the governing IRBs of the ECHO trial to make de-identified publicly available.
