# Supplementary material for: Sexually transmitted infections among women randomised to depot medroxyprogesterone acetate, a copper intrauterine device or a levonorgestrel implant
Source: Sex Transm Infect. 2020 Nov 18;97(4):249–55. doi: 10.1136/sextrans-2020-054590 (PMC8165154; doi:10.1136/sextrans-2020-054590)
Supplement: Supplementary data [file sextrans-2020-054590supp001.pdf]

Appendix 1: Table of clinical assessments and findings

	DMPA-IM n (%)	Copper IUD n (%)	LNG implant n (%)
Month 3			
Pelvic exams performed	357 (14)	665 (26)	432 (17)
Abdominal/pelvic pain on exam	30/357 (8)	167/665 (25)	54/432 (13)
Discharge observed[a]	69/357 (19)	208/665 (31)	103/432 (24)
Month 6			
Pelvic exams performed	287 (12)	539 (22)	355 (14)
Abdominal/pelvic pain on exam	36/287 (13)	147/539 (27)	38/355 (11)
Discharge observed[a]	57/287 (20)	181/539 (34)	74/355 (21)
Month 9			
Pelvic exams performed	312 (13)	495 (20)	363 (15)
Abdominal/pelvic pain on exam	51/312 (16)	131/495 (27)	53/363 (15)
Discharge observed[a]	69/312 (22)	199/495 (40)	86/363 (24)
Month 12			
Pelvic exams performed	481 (21)	683 (28)	589 (24)
Abdominal/pelvic pain on exam	43/481 (9)	140/683 (21)	43/589 (7)
Discharge observed[a]	56/481 (12)	184/683 (27)	101/589 (17)
Month 15			
Pelvic exams performed	650 (32)	782 (36)	721 (33)
Abdominal/pelvic pain on exam	40/650 (6)	127/782 (16)	50/721 (7)
Discharge observed[a]	66/650 (10)	159/782 (20)	84/721 (12)
Month 18			
Pelvic exams performed	1679 (100)	1738 (100)	1754 (100)
Abdominal/pelvic pain on exam	34/1679 (2)	59/1738 (3)	30/1754 (2)
Discharge observed[a]	86/1679 (5)	163/1738 (9)	104/1754 (6)

DMPA-IM=intramuscular depot medroxyprogesterone acetate. IUD=intrauterine device. LNG=levonorgestrel.

[a] Abnormal result showing vaginal discharge, mucopurulent discharge and/or other cervical discharge.
